# HELQ & Hel308: ancient enzymes of DNA repair and recombination

**DOI:** 10.1042/BST20250255

**Published:** 2026-09-29

**Authors:** Anna Lou-Hing, Olivia Downs, Thorsten Allers, Edward L. Bolt

**Affiliations:** The School of Life Sciences, University of Nottingham, Nottingham, U.K.

**Keywords:** DNA repair, helicase, recombination

## Abstract

We highlight the multifunctionality of the Hel308 and HELQ Ski2-like ‘helicases’ as they toggle between DNA annealing and DNA translocation across multiple DNA repair pathways. Defects in Hel308/HELQ cause genetic instability, including dysregulated homologous recombination. New data reveal how Hel308 and HELQ switch between DNA translocation and annealing, a mechanism that may restrain recombination to short tracts by curbing extensive DNA synthesis. We describe a PWI-like innovation in HELQ that evolved in bilaterian animals—absent from other animals and archaea—which we propose is an ATR-targeted phosphoregulatory module, coordinating protein interactions and, through those, DNA repair-recombination reactions. The overall picture is of Hel308 and HELQ as ancient DNA annealing enzymes, aided by short bursts of ATP-powered DNA translocation, kept in balance by molecular switches and the additional regulatory surfaces that arose in some animals.

## The origins of HELQ and Hel308

Helicases are powered by ATP hydrolysis at a phosphate-binding loop (P-loop) embedded in either a RecA or an AAA^+^ protein domain, a distinction that is part of helicase classification into superfamilies 1–6 [1,2]. P-loops were present in LUCA (‘Last Universal Common Ancestor’) [3,4], diverging into the RecA and AAA^+^ lineages [5]. This duality is apparent within replicative helicases, which require a RecA fold in bacteria (DnaB [6]) or an AAA^+^-fold in archaea (MCM [7]) to split parental DNA duplexes into a replication fork. Nonetheless, both RecA and AAA^+^ proteins are characterised by conservation of ATP binding (Walker A) and hydrolysis (Walker B) motifs within the P-loop [8].

The RecA fold was first observed in the RecA recombinase protein [9], which catalyses DNA strand exchange reactions that create D-loops, initiating homologous recombination in bacteria—structural and functional homologues of RecA are RadA and Rad51 in archaea and eukaryotes, respectively. A few years later the first helicase structure (PcrA [10]) showed two RecA-like domains (1A and 2A) of four domains in total, establishing what is now known to be common: tandem RecA1 and RecA2 domains that bind to and hydrolyse ATP whilst gripping nucleic acid strands. This powers cycles of RecA conformational change that translocates DNA strands and may unwind DNA duplexes in that process. The RecA architecture was predicted, using bioinformatics, before being visualised [11], and revealed commonality in helicase mechanism [12], and a recent biography of helicase discovery can be found in [13].

‘Core’ RecA helicase/translocase domains are accessorised with folds and domains that provide specific functions in DNA and RNA processing. The HELQ and Hel308 Ski2-like helicases exemplify this [14–16] (Figure 1), comprising five domains; the RecA1–RecA2 core translocase domains 1 and 2, accessorised with a winged helix domain 3 (substrate recognition [17]), a ‘ratchet’ domain 4 for traversing single-stranded DNA (ssDNA), and a helix-hairpin-helix domain 5 (DNA annealing). Additionally, HELQ has a ∼300 amino acid extension at its N-terminus (“N-HELQ”) that comprises an intrinsically disordered protein region (IDPR, residues ∼1–130) and a predicted four-helical PWI-like fold that is most similar to the Ski2 Brr2 protein [18] (residues ∼130–240). Members of the Ski2-like helicase group are found across bacteria, archaea and eukaryotes, in archaea splitting into Hel308-like and ASH-like branches [19,20]—archaeal Hel308 is sometimes instead called Hjm (for ‘Holliday junction migration’), based on a biochemical activity described in [21], discussed in [22,23]. The Hel308 protein is present in Asgard archaea and in choanoflagellates—the closest unicellular ancestors of animals [24]—consistent with an archaeal origin of eukaryotes [25–28] and the later acquisition of the N-HELQ region in bilaterian animals. However, although the Brr2 Ski2-like proteins are present throughout fungi, Hel308/HELQ proteins are not.

**Figure 1:**
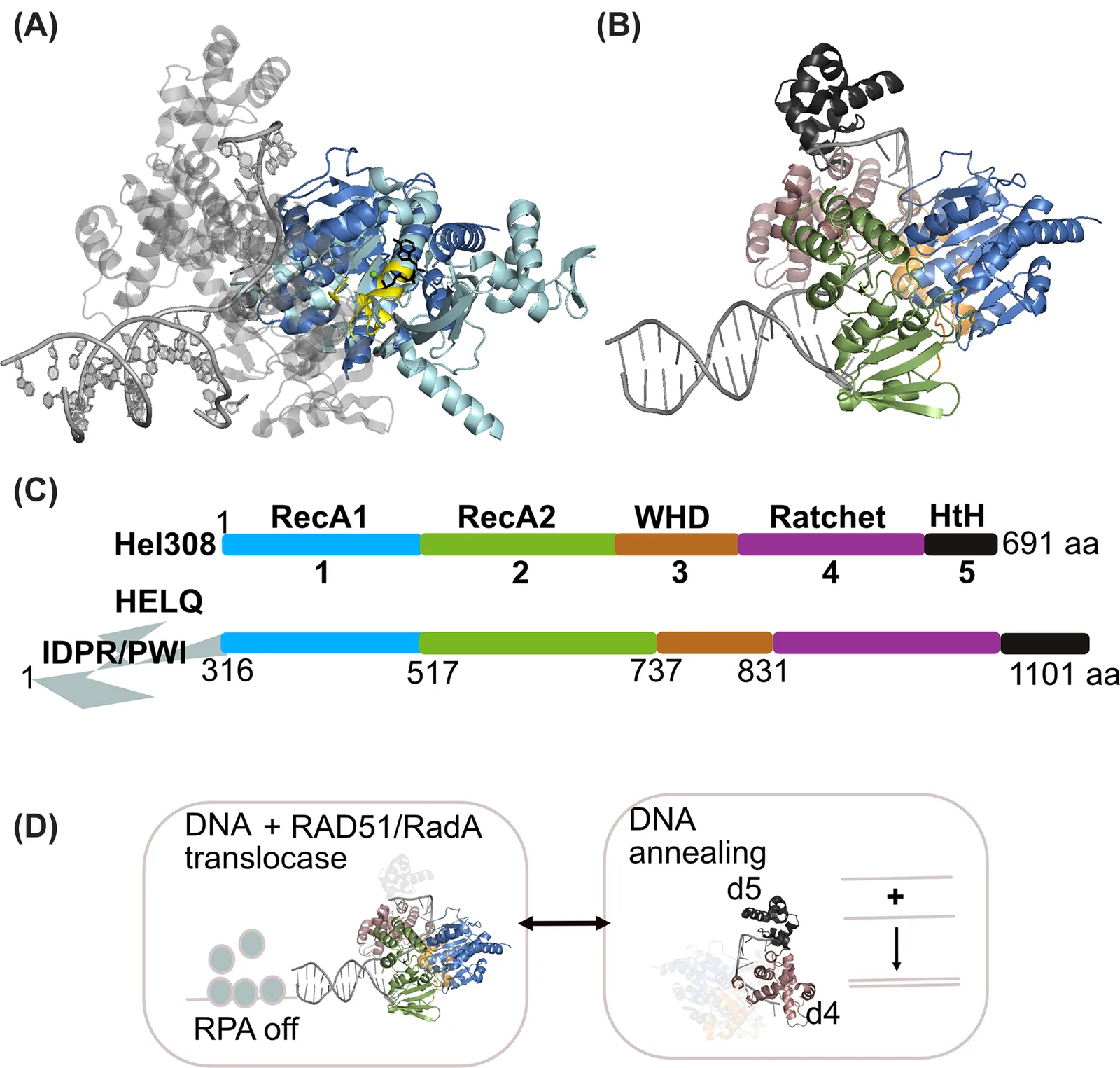
Conserved architecture of the Hel308/HELQ helicase–annealase and its bilaterian addition “N-HELQ” (**A**) The shared RecA fold of the RecA1 domain of *Archaeoglobus fulgidus* Hel308 (blue; PDB 2P6R) superposed with *Escherichia coli* RecA (cyan; PDB 2REB). The remainder of Hel308 (domains 2–5) and bound DNA are faded grey. The P-loop (Walker A motif) is shown in yellow with bound unhydrolysable ATP in black. (**B**) Hel308 (PDB 2P6R), coloured by domain and bound to a partly unwound DNA duplex: RecA1 (1, marine), RecA2 (2, smudge green), winged-helix domain (3, WHD, orange), ratchet (4, dirty pink), and the C-terminal helix-hairpin-helix domain (5, near black), which are represented in the same colours in panel (**C**), which highlights conservation of the five-domain core from archaeal Hel308 (amino acids 1–650) to HELQ (amino acids 300–1100)—bilaterian animals have an added N-terminal region that is absent from archaeal Hel308 (N-HELQ). N-HELQ comprises an intrinsically disordered region plus a predicted PWI-like fold, IDPR/PWI. (**D**) Hel308 and HELQ interconvert DNA from single-stranded to double-stranded forms (double arrow). Left: domains 1–4 provide ATP-powered DNA translocation that strips RPA from ssDNA, stimulated by a RAD51/RadA protein contact. Right: domains 4 and 5 switch the enzyme to DNA annealing, pairing complementary ssDNA into duplex.

## One motor, two activities: DNA translocase and annealase in genome stability

Hel308 and HELQ are ssDNA-dependent ATPases that translocate with 3′ to 5′ directionality, and they can unwind duplex DNA [21,23,29,30]; their discovery is reviewed in [31]. Hel308 and HELQ helicase activity—separation of DNA strands—decreases sharply to 2% of the total DNA becoming unwound when duplex length is increased from 20 to 80 base pairs, and a ssDNA (i.e. ‘tailed’) region is needed for duplex unwinding, in line with the ssDNA-triggered ATPase activity [14,23,29]. HELQ/Hel308 monomers or dimers appear to be helicase-active, with tetramers or hexamers possibly formed in non-helicase conditions [18,29].

Translocation of ssDNA by Hel308 is observable at sub-nucleotide resolution through MspA protein nanopores [32], showing processivity—nucleotides translocated before enzyme dissociation—of about 1 knt [33]. Hel308 steps twice per nucleotide and also backsteps (i.e. 5′ to 3′) depending on whether ATP or ADP is bound, with backstepping and dwell time—how long it is stationary before stepping—influenced by specific bases, which form contacts with residues conserved in the RecA domains of archaeal Hel308 and metazoan HELQ [34–36]. Therefore Hel308 is processive at translocating but not unwinding DNA duplexes, consistent with, detailed below, the stripping of ssDNA-binding protein RPA from DNA to facilitate annealing of complementary DNA strands. In this activity, annealing RPA-bound DNA, Hel308/HELQ join the family of ‘annealases’ HARP/SMARCAL1 [37,38], RAD52 [39], and DNA polymerase θ [40–42].

Control of archaeal Hel308 helicase activity was suggested from the *Archaeoglobus* Hel308 structure bound to a partial DNA duplex [15], which includes the helix-hairpin-helix domain 5 bending ssDNA in a dog’s-leg shape, positioned away from the core helicase and ratchet domains (Figure 1). Mutations and truncations of Hel308 domain 5 revealed that it is a brake on helicase and ATPase activity, as a sensor of ssDNA [14,43], although deletion of domain 5 had no significant effect on DNA translocation by Hel308 through nanopores [36]. Cryo-EM structures of the polymerase θ Hel308-like helicase region revealed that its orthologous domain 5 is a ‘gate’ that swings outwards in response to ssDNA binding, aligning two DNA strands within a protein dimer for strand annealing [41,42]. This flexibility relies on a swivelling alpha-helix that connects domains 4 and 5—and which is well conserved in archaeal Hel308 and HELQ—that moves and re-orders the RAD51-interacting surface of polymerase θ, perhaps mechanistically coupling DNA annealing with regulation of homologous recombination. HELQ physically interacts with Rad51 (next section), and it has been suggested that Hel308 interacts with RadA [30], though clear data for that is lacking.

The RecA1-RecA2 core may also contribute to control of annealing, as revealed by a striking single amino acid substitution (Hel308 Phe-295-Ala and HELQ Tyr-642-Ala) in the RecA2 domain motif IVa, which results in both Hel308 and HELQ being hyperactive at DNA annealing and DNA binding ([44] and unpublished). Molecular dynamics simulations suggest this residue change orientates RecA2 into a more open conformation [44], which could stimulate annealing by either prolonging occupancy of ssDNA, accelerating DNA strand nucleation, or repositioning domains 4 and 5 into a favourable position for ssDNA annealing. In support of the latter, unpublished biochemical data show that a purified Hel308/HELQ domain 4–5 polypeptide, lacking the RecA domains, is a hyperactive annealase, indicating wild-type RecA domains may be a brake on annealing. Whatever the precise cause, the cellular effect of this single amino acid substitution was startling—gene conversion increases by 166 000-fold, yet DNA cross-link repair is unaffected compared with wild-type cells [44], separating Hel308’s recombination-suppressing role from any direct DNA repair role.

Loss of Hel308 or HELQ destabilises genomes in archaea and animals alike: cells become hypersensitive to DNA cross-linking agents [44–52] and are defective in single-strand annealing (SSA) and microhomology-mediated end joining (MMEJ) [50,53,54]. The effect of their loss on homologous recombination is dysregulation, not failure—RAD51 foci and filaments persist, recombination frequency changes, and gene-conversion tracts lengthen [44,45,51,53]—indicating that Hel308 and HELQ limit recombination by DNA replication after DNA strand exchange. These phenotypes, together with the biochemical activities of Hel308/HELQ, indicate control of DNA annealing within multiple arms of DNA repair, distinct from another major pathway of DNA annealing by RAD52 (detailed below). Genome-wide CRISPR and RNAi screens of ∼1150 cancer cell lines make clear that loss of HELQ has essentially no effect on cell proliferation as a non-essential gene, clustering near 0, versus ≈ −1 for pan-essential genes (DepMap/Project Achilles [55]). This is consistent with the indirect effects of HELQ on causes or progress of cancers, through a generalist role in maintaining genome stability by DNA annealing.

In mice, HELQ loss causes subfertility and ovarian tumorigenesis [45], and in human ovarian cancers, HELQ copy number losses may be similar to the RAD51 paralogues, marking it as a candidate susceptibility gene [46]. However, elevated HELQ expression in such tumours tracks with cisplatin resistance and poor prognosis, so the protein is tumour-suppressive when lost but a marker of poor outcome in excess [56–58]. Lengthening of recombination tracts when HELQ is defective promotes “genome instability and mutation”, a defined enabling characteristic of cancers [59]. This is because lengthened gene-conversion tracts promote loss of heterozygosity that may unmask recessive mutations, and reflects mutagenic DNA synthesis that is characteristic of cells that must survive replication stresses through recombination by break-induced replication (BIR) [60–63].

## Switching between annealing and unwinding: learning from biochemistry

The switch mechanism between DNA annealing and translocase functions is central to understanding how Hel308/HELQ controls multiple DNA repair paths. Firstly, interaction of HELQ with RPA stimulates DNA annealing by recruiting HELQ to ssDNA, triggering its ssDNA- and ATP-dependent DNA translocase activity, which strips RPA from DNA non-processively [53]. Once this is done, the resulting DNA strand pairing does not need ATP—an ATPase-null HELQ mutant can anneal naked DNA strands, but it cannot anneal RPA-bound DNA [53]. A similar process is likely for archaeal Hel308, which also interacts with RPA [64], even though Hel308 lacks the N-IDPR of HELQ. The molecular details of the Hel308–RPA and HELQ–RPA interactions remain unresolved, except that the predicted PWI-like fold within N-HELQ region is required ([18], and authors’ unpublished NMR data), a region lacking in archaeal Hel308 proteins, as discussed in the final section.

Secondly, Rad51–HELQ interaction stimulates HELQ helicase activity by protein–protein contact—neither DNA binding nor filament formation by Rad51 was needed, and bacterial RecA cannot substitute for RAD51 [53]. This makes it unlikely that the complex acts to clear RAD51 from D-loops, especially considering that HELQ is poor at stripping RAD51 protein from nucleoprotein filaments [53], although *C. elegans* HELQ removes RAD51 from DNA duplexes [51]. Perhaps interaction of HELQ and RAD51 in the absence of DNA delivers HELQ to sites of recombination—for example, at the leading edge of an advancing D-loop as RAD51 is displaced and DNA-free. RAD51-stimulated DNA unwinding by HELQ (after loading by RPA) may then dissociate duplexed nascent and template DNA strands in the D-loops, favouring re-annealing and termination of synthesis, rather than the extended tracts generated from unchecked BIR.

Thirdly, HELQ physically and functionally interacts with the POLD3 subunit of the polymerase δ (delta), inhibiting its DNA synthesis [65]. This inhibition is independent of DNA binding by HELQ and is effective against the Pol δ–RPA–PCNA holo-complex, ruling out HELQ acting simply as a physical block to primer extension. Instead, the N-terminal IDPR region of HELQ was responsible for this effect, an interaction that stimulates DNA annealing by the HELQ core domains [65]. Because polymerase δ drives mutagenic DNA synthesis within D-loops during BIR, its inhibition by HELQ may limit the extent of this type of recombination and, therefore, mutagenesis, consistent with increased long-tract gene conversion when HELQ is lost [53]. In cancer cells, the BIR-like process MiDAS needs RAD52 for DNA annealing [65] and a sequential handoff from the polymerase Pol ζ (zeta) to Pol δ [66], both of which need a POLD3 subunit. However, untransformed cells instead perform MiDAS independently of RAD52, in a process requiring HELQ [67]. It is tempting to propose that HELQ governs this process in healthy cells through its interaction with POLD3—favouring Pol ζ over Pol δ, and substituting for RAD52 as the annealase. HELQ/Hel308 is thought to anneal DNA strands during SSA and MMEJ [50,53,54], which pair imperfectly matched DNA sequences. Further, mutagenic DNA synthesis by polymerase δ within D-loops generates nascent strands mismatched with template and sister chromatid; whether HELQ/Hel308 at least tolerates these or is biased against them would indicate whether this DNA annealing halts error-prone DNA synthesis or consolidates it.

Core translocase-helicase functions of archaeal Hel308 are likely to be similar to HELQ in archaeal recombinational repair, but the context of action is less clear. In archaea, recombination-dependent DNA replication-repair independent from origins of replication requires RadA [68], and this process has been reconstituted *in vitro* using RadA and polymerases [69]. Hel308 interacts physically with RPA [64] and possibly RadA [30]; the interaction with RPA raises the possibility of competition between Hel308 and replisome assembly by the archaeal primase (PriSL) and the archaeal replicative polymerase PolD (distinct from eukaryotic POLD/Pol δ)—both of which interact with archaeal RPA [70]—perhaps favouring RadA triggered recombination-replication. Hel308 also interacts with the archaea-specific recombination enzyme Hjc [71] and the archaeal PIN-superfamily protein PINA [72], the latter intriguing because human HELQ also interacts with a PIN protein, the XRN2 nuclease [73]. All of this indicates Hel308 as a regulator of DNA repair and recombination through multiple protein–DNA interactions.

## Protein interactions position Hel308 and HELQ in cells

In animals, both HELQ and RAD52 contribute to genome stability by DNA annealing but by divergent mechanisms—RAD52 is lucidly described in [39]. Both contribute to multiple DNA repair pathways: for SSA, depletion or deletion of HELQ and RAD52 is epistatic—indicates the same pathway—but is additive for gene conversion by recombination [53]. However, these distinct pathways both involve RPA and RAD51: RPA interactions provide access to ssDNA for Hel308/HELQ annealing reactions, and interactions with RAD51/RadA may provide entry to modulate specifically homologous recombination.

In addition to RPA and RAD51, HELQ physically interacts with the RAD51 paralogue complex BCDX2 (RAD51B, -C, and -D and XRCC2) [46]. The RAD51 paralogue complexes more broadly regulate the extent of gene conversion tract length during recombination—cells lacking them are biased towards long tract gene conversion [74–76], a phenotype similar to loss of HELQ. HELQ-paralogue physical interaction may indicate they co-operate to limit gene conversion tracts, at least in some contexts.

The relationship between HELQ and HROB-MCM8-MCM9 helicase complex is also revealing about control of gene conversion tract length. The additive effect on the severity of recombination defects from deleting both HELQ and HROB-MCM8-MCM9 [77] indicates that they act in different pathways to control recombination. Further, they seem to act in opposite directions: MCM8-MCM9 helicase, loaded by the HROB protein, promotes DNA synthesis post-RAD51 synapsis—analogous to the helicase Pif1 in some yeasts—driving long tract DNA synthesis [78], whereas HELQ limits this. The two are therefore antagonistic over gene-conversion tract length, setting a balance between short- and long-tracts. How a cell tips this balance toward one or other—whether by DNA substrate, cell-cycle stage, chromatin, or availability of POLD3 and RAD51 paralogues—remains unknown.

## N-HELQ: a recently evolved HELQ interaction site influencing recombination

HELQ and Hel308 in animals and archaea have the same five helicase-annealase domains, but a ∼300-residue N-terminal extension (“N-HELQ”) arose in just one of the five animal groups, Bilateria, around 550+ million years ago (Figure 2). The four other major animal groups (Placozoa, Ctenophora, Porifera, and Cnidaria) all encode the protein lacking N-HELQ, equivalent to archaeal Hel308. Rather than adding any catalytic capacity—N-HELQ has no detectable DNA-binding activity [18,65]—the region provides regulatory controls for HELQ function.

**Figure 2:**
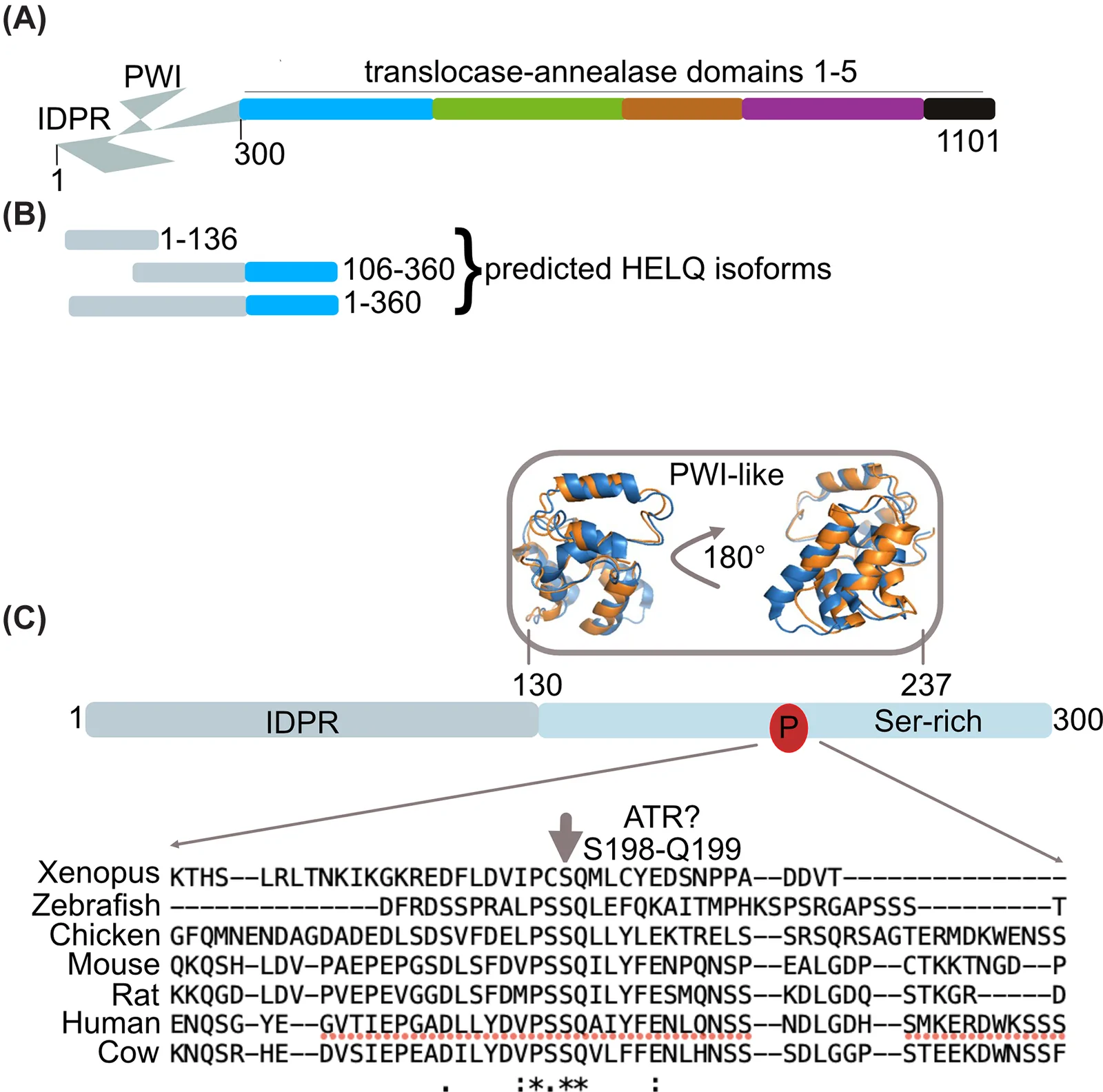
The N-terminal N-HELQ region: context and predictions (**A**) Domain organisation of human HELQ showing the conserved translocase–annealase core (domains 1–5; residues ∼300–1101) preceded by the bilaterian-specific N-HELQ region, which can be subdivided into a disordered segment (IDPR) and a predicted PWI-like fold. (**B**) Three predicted human HELQ isoforms (residues 1–136, 1–360, and 106–360) that may encode the N-HELQ region, raising the possibility that N-HELQ is expressed independently of the catalytic core. (**C**) The N-HELQ region (residues 1–300), highlighting the predicted PWI-like fold [18] (∼residues 130–237) shown as a structural superposition (blue) on the ‘non-canonical’ PWI domain (orange) experimentally solved for the Ski2 protein Brr2 [84]. Two views are given to emphasise close structural superimposition. A candidate ATR kinase phosphorylation target site at Ser198–Gln199 (red marker) is within the serine-rich tract (residues 192–270, lilac). Below this, a Clustal Omega multiple-sequence alignment shows conservation across diverse bilaterians of the Ser–Gln as indicated. Residues are marked * (identical), (strongly similar), or (weakly similar).

### A separable, semi-autonomous regulatory module

An N-HELQ protein fragment (comprising HELQ amino acids 1–240) was first isolated by accident, co-purifying with full-length HELQ after expression of the *HELQ* gene in Sf9 insect cells [18]. It comprises two major parts—a predicted PWI-like fold [18] (residues ∼ 130–240) and the IDPR (1–130), which is unusually rich in polar, acidic and basic amino acid residues (Figure 2). N-HELQ is separable from the helicase-annealase domains 1–5 of HELQ (‘C-HELQ’) without any apparent loss of C-HELQ ATPase, helicase or annealase activity [18]. Consistent with a semi-autonomous regulatory role for N-HELQ, three of the four predicted human HELQ isoforms identified from transcriptomics studies [79,80] are predicted to encode peptides comprising residues 1–136, 1–360, and 106–360. This raises the speculative possibility that N-HELQ polypeptides may be expressed alone to act in *trans* as a stand-alone regulatory protein.

N-HELQ provides a site for interaction with RPA and DNA polymerase delta. It, when acting alone as a purified fragment or in HELQ, binds to both. The interaction with RPA involves two residues (Asp-142 and Phe-143 in human N-HELQ region) that are situated in one of the predicted PWI-like helices and are required to destabilise RPA–ssDNA complexes [18]. N-HELQ interacts with the POLD3 subunit of DNA polymerase delta in the third IDPR region of POLD3 (unpublished) halting DNA synthesis by the polymerase holoenzyme and stimulating the HELQ DNA annealing reaction. Archaeal Hel308 also interacts with RPA through a conserved motif at its C-terminus [64]. Therefore, it appears that RPA-dependent delivery of Hel308/HELQ to ssDNA has been solved at opposite ends of the protein in bilaterians and archaea.

### Phospho-regulation of HELQ by ATR at N-HELQ?

Proteomic datasets for HELQ [81] detect multiple phosphorylation sites (most map to the N-terminal region), and two acetylation and two ubiquitination sites (all in annealase-helicase domains 1–5). These have obvious potential for further regulation of HELQ’s protein interactions or annealase-helicase activities, but there is not yet supporting experimental data.

However, amino acid sequence alignments raise a couple of intriguing possibilities. Firstly, a serine-rich cluster in N-HELQ is apparent from residues 195–270 (using numbering from human N-HELQ) (Figure 2). Overall serine amino acid content that is across all of human HELQ (7.8%) rises to 18% in these N-HELQ residues, which may be significant as a multi-site phosphorylation module modifiable for protein function, or for controlling liquid–liquid phase condensation, or for PEST-like proteolysis to allow turnover of proteins that need rapid regulation/de-regulation, all of which are associated with serine-rich tracts.

Secondly, and more specifically, at the beginning of this serine tract is a Ser-198/Gln-199 pair (using the human HELQ residue numbers)—in an overall motif (V/M)P-S-S-Q-(A/I/V)(I/L)—that is conserved across mammals, frogs, and fish, something that is overall highly unusual for residues across N-HELQ. This SQ matches the consensus phosphorylation site for ATM/ATR family kinases [82,83], its significance is made more convincing because of physical interaction of HELQ with ATR [46]. HELQ serine-198 is not recorded as phosphorylated in proteome database, which may be explained by it being ‘hidden’ within a long (47 amino acids) lysine/arginine-poor tryptic peptide. The phosphosites that have been detected all fall within clustered lysine/arginine-flanked serines that trypsin cleaves cleanly, so the phosphorylation outputs probably reflect peptide detectability as much as true sites, leaving some conserved but digestion-refractory residues (e.g. Ser198) unrecorded. Phosphorylation of HELQ by ATR may also depend on a DNA-damage or replication-stress response, conditions under which the cited proteomic data were not collected. We propose N-HELQ as a candidate phosphoregulatory module for coordinating HELQ across multiple DNA-repair pathways.

## Overall conclusion

The picture that emerges is of HELQ/Hel308 as a DNA annealase facilitated by DNA translocation and displacement of RPA from DNA, controlled internally—domain 5 and the RecA2 motif IVa—and allosterically by interacting proteins. HELQ is more a controller of recombination than a direct DNA repair enzyme: its loss triggers genetic instability characteristic of unrestrained mutagenic DNA synthesis during recombination-repair. An open question is, what is the fidelity of DNA annealing by Hel308/HELQ? Error-prone DNA synthesis during, for example, BIR in animals generates clusters of mismatched DNA within longer homologous DNA strands, a process driven by DNA polymerase δ, whose POLD3 subunit physically and functionally interacts with HELQ [65]. Bias against pairing of mismatched DNA strands by Hel308/HELQ may indicate checkpoint control to prevent further error prone synthesis, fitting with genetic instability when Hel308 (HELQ-1) is deleted in *C. elegans* [50]. Further, the 5–10 amino acid swivelling hinge region of domain 5, which seems to be crucial for annealing DNA, may be a target for small molecule inhibitors.

Bilaterian metazoans control HELQ by non-catalytic actions of the N-HELQ region: a 300 amino acid extension of two parts, a disordered region and a serine-rich PWI-like fold. N-HELQ appears to physically connect the core enzyme annealase-translocase to RPA and Pol δ in animals, and perhaps to other protein partners. The circumstantial evidence that N-HELQ is phospho-regulated by ATR is a clear and open question for further study, and one that may offer a route to disrupt that control. A full-length HELQ structure resolving N-HELQ against the motor would be informative, as would knowing whether N-HELQ acts *in trans* in cells, as its *in vitro* activities and predicted isoforms hint may be the case. Archaeal Hel308 may be valuable for understanding details of the DNA annealing mechanism and recombination in archaea more broadly, especially through genetics and biochemistry utilising mutations in domain 5 and the hyper-recombinogenic RecA2 site.

## Perspectives

- *Significance:* Hel308/HELQ proteins are conserved from archaea to humans, where they support multiple DNA repair and recombination pathways. Their failure triggers long tract gene conversion and mutagenesis—genome instability that is an enabling characteristic of cancers.
- *Updated thinking:* Hel308/HELQ are less ‘helicases’ and more DNA annealing enzymes, using bursts of ATP-dependent DNA translocation to displace DNA-bound proteins and drive DNA strand annealing. Their interactions with DNA repair and recombination enzymes may control the extent of DNA synthesis during DNA repair, and so shape the extent of mutagenesis in cells.
- *Future directions:* We propose that the 300-amino acid extension to HELQ (“N-HELQ”) in bilaterian animals is a phosphoregulatory hub targeted by ATR kinase, co-ordinating HELQ to anneal DNA. Understanding that, and the core DNA annealing mechanism shared by Hel308 and HELQ, will reveal precisely how they control DNA repair and recombination.

## Open Access

Open access for this article was enabled by the participation of University of Nottingham in an all-inclusive Read & Publish agreement with Portland Press and the Biochemical Society under an agreement with JISC.

## Abbreviations

BIR: break-induced replication
MMEJ: microhomology-mediated end joining
P-loop: phosphate-binding loop
SSA: single-strand annealing
ssDNA: single-stranded DNA
